# Single Cell Isolation and Analysis

**DOI:** 10.3389/fcell.2016.00116

**Published:** 2016-10-25

**Authors:** Ping Hu, Wenhua Zhang, Hongbo Xin, Glenn Deng

**Affiliations:** ^1^The Center for Biotechnology and Biopharmaceutics, Institute of Translational Medicine, Nanchang UniversityNanchang, China; ^2^Laboratory of Fear and Anxiety Disorders, Institute of Life Science, Nanchang UniversityNanchang, China; ^3^Yichang Research Center for Biomedical Industry and Central Laboratory of Yichang Central Hospital, Medical School, China Three Gorges UniversityYichang, China; ^4^Division of Surgical Oncology, Stanford University School of MedicineStanford, CA, USA

**Keywords:** heterogeneity, single cell, isolation, analysis, sequencing

## Abstract

Individual cell heterogeneity within a population can be critical to its peculiar function and fate. Subpopulations studies with mixed mutants and wild types may not be as informative regarding which cell responds to which drugs or clinical treatments. Cell to cell differences in RNA transcripts and protein expression can be key to answering questions in cancer, neurobiology, stem cell biology, immunology, and developmental biology. Conventional cell-based assays mainly analyze the average responses from a population of cells, without regarding individual cell phenotypes. To better understand the variations from cell to cell, scientists need to use single cell analyses to provide more detailed information for therapeutic decision making in precision medicine. In this review, we focus on the recent developments in single cell isolation and analysis, which include technologies, analyses and main applications. Here, we summarize the historical background, limitations, applications, and potential of single cell isolation technologies.

## Introduction

The cell is the fundamental unit of biological organisms. Despite the apparent synchrony in cellular systems, analyzed single cell results show that even the same cell line or tissue, can present different genomes, transcriptomes, and epigenomes during cell division and differentiation (Schatz and Swanson, 2011). For example, a developing embryo, brain, or tumor have intricate structures consisting of numerous types of cells that may be spatially separated. Thus, the isolation of distinct cell types is essential for further analysis and will be valuable for diagnostics, biotechnological and biomedical applications.

Conventional cell-based assays mainly measure the average response from a population of cells, assuming the average response is representative of each cell. However, in doing this important information about a small but potentially relevant subpopulation maybe lost, particularly in cases where that subpopulation determines the behavior of the whole population. For example, the tumor microenvironment is a complex heterogeneous system that consists of multiple intricate interactions between tumor cells and its neighboring non-cancerous stromal cells. The stromal cells are composed of endothelial cells, fibroblasts, macrophages, immune cells, and stem cells. Due to the variation in genetic and environmental factors, different kinds of cells have unique behaviors and present different implications in pathogenic conditions (Schor and Schor, 2001). These challenges make conventional analysis insufficient. Therefore, new technologies to isolate individual single cells from a complex sample and study the genomes and proteomes of single cells could provide great insights on genome variation and gene expression processes. It is believed that single cell analyses have influences on various fields including life sciences and biomedical research (Blainey and Quake, 2014).

In early times, researchers have applied low-throughput single cell analysis techniques, such as immunofluorescence, fluorescence *in situ* hybridization (FISH) and single cell PCR, to detect certain molecular markers of single cells (Taniguchi et al., 2009; Citri et al., 2012). These techniques allow quantification of a limited number of parameters in single cells. On the other hand, high-throughput genomic analysis, such as DNA and RNA sequencing are now widely used. However, genomic studies rely on studying collective averages obtained from pooling thousands to millions of cells, precluding genome-wide analysis of cell to cell variability. Therefore, single cell sequencing developed alongside its necessity in research awarding it “method of the year” by Nature Methods in 2013 (2014). By using single cell analysis, researchers have profiled many biological processes and diseases at the single cell level including tumor evolution, circulating tumor cells (CTCs), neuron heterogeneity, early embryo development, and uncultivatable bacteria.

In this review, we discuss the technologies recently developed for single cell isolation, genome acquisition, transcriptome, and proteome analyses, and their applications. We also briefly discuss the future potentials of single cell isolation technologies and analyses.

## Technologies for single cell isolation

Before initiating a single cell analysis, scientists need to isolate or identify single cells. The performance of cell isolation technology is typically characterized by three parameters: efficiency or throughput (how many cells can be isolated in a certain time), purity (the fraction of the target cells collected after the separation), and recovery (the fraction of the target cells obtained after the separation as compared to initially available target cells in the sample). The current techniques show different advantages for each of the three parameters.

Based on the variety of principles used, current existing cell isolation techniques can be classified into two groups. The first group is based on physical properties like size, density, electric changes, and deformability, with methods including density gradient centrifugation, membrane filtration and microchip-based capture platforms. The most advantageous physical properties is single cell isolation without labeling. The second group is based on cellular biological characteristics, comprising of affinity methods, such as affinity solid matrix (beads, plates, fibers), fluorescence-activated cell sorting, and magnetic-activated cell sorting, which are based upon biological protein expression properties (Dainiak et al., 2007). Thus, in what follows we briefly summarize the principle of each method, as well as the advantage and limitation of their applications (Table 1). We will not discuss limiting dilution since it is well known in the field of monoclonal cell cultures production.

**Table 1:** **Overview of single cell isolation techniques**.

**Techniques**	**Throughput**	**Advantage**	**Disadvantage**	**References**
Fluorescence-activated cell sorting (FACS)	High	High specificity multiple parameters	Large amount of material, dissociated cells, high skill needed	Gross et al., 2015
Magnetic-activated cell sorting (MACS)	High	High specificity, cost effective	Dissociated cells, non-specific cell capture	Welzel et al., 2015
Laser capture microdissection (LCM)	Low	Intact fixed and live tissue	Contaminated by neighboring cells, high skill needed	Espina et al., 2007; Datta et al., 2015
Manual cell picking	Low	Intact live tissue	High skill needed, low throughput	Citri et al., 2012
Microfluidic	High	Low sample consumption, integrated with amplification	Dissociated cells, high skill needed	Bhagat et al., 2010; Lecault et al., 2012

### Fluorescence activated cell sorting (FACS)

**Figure 1:**
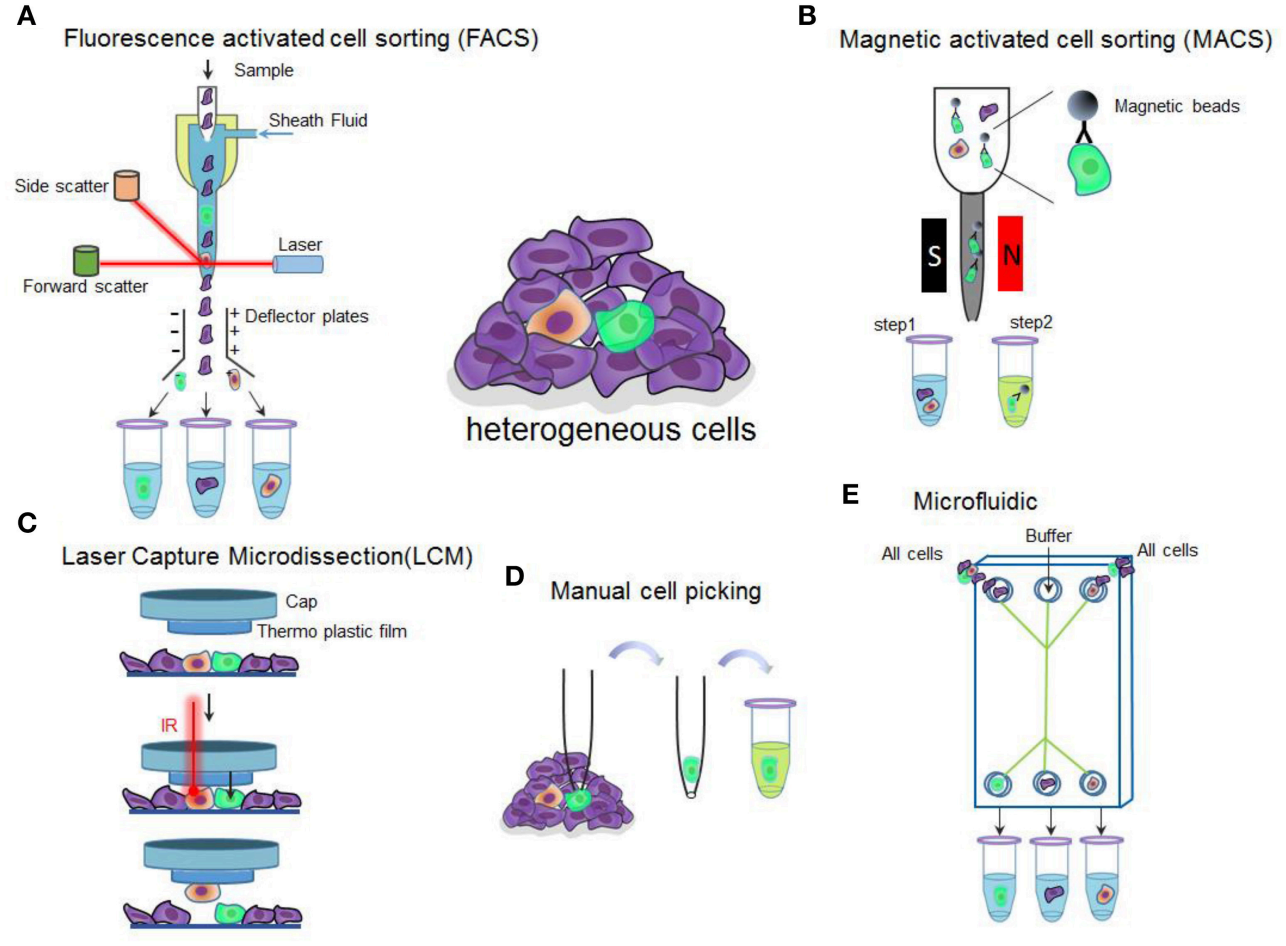
**Overview of single-cell isolation technologies discussed in the section. (A)** Schematic of fluorescence-activated cell sorting. The suspended labeled cells are passed as a stream in droplets with each containing a single cell in front of a laser. The fluorescence detection system detects the fluorescent and light scatter characteristics. Based on their characteristics, the instrument applies a charge to the droplet containing a cell of interest and an electrostatic deflection system facilitates collection of the charged droplets into different collecting tubes. Cells labeled with green, purple, and yellow indicate different cell types. **(B)** Schematic of magnetic-activated cell sorting. Cells of interest are labeled with specific antibody conjugated magnetic beads. An external magnetic field is used to separate the labeled cells from the cell suspension. S and N indicate magnetic field. **(C)** Schematic of laser capture microdissection. The technique utilizes a laser which fired through the cap over the cells of interest to melt the membrane to let the cells adhere to the melted membrane. When the cap is removed, captured cells are removed, leaving the unwanted cells behind. **(D)** Schematic of manual cell picking. The cells of interest are monitored under a microscope. By using a glass pipette connected to a micromanipulator, single cells can be collected and transferred to a new tube for following analysis. **(E)** Schematic of microfluidic used for single cell isolation. Before starting the experiments, cells need to be dissociated then flow into a chip. Thus, the cells may be separated into different tubes containing only one cell.

Fluorescence Activated Cell Sorting (FACS), a specialized type of flow cytometry with sorting capacity, is the most sophisticated and user-friendly technique for characterizing and defining different cell types in a heterogeneous cell population based on size, granularity, and fluorescence. FACS allows simultaneous quantitative and qualitative multi-parametric analyses of single cells (Gross et al., 2015). Before separation, a cell suspension is made and the target cells are labeled with fluorescent probes. Fluorophore-conjugated monoclonal antibodies are the most widely used fluorescent probes (mAb) that recognize specific surface markers on target cells. As the cell suspension runs through the cytometry, each cell is exposed to a laser, which allows the fluorescence detectors to identify cells based on the selected characteristics. The instrument applies a charge (positive or negative) to the droplet containing a cell of interest and an electrostatic deflection system facilitates the collection of the charged droplets into appropriate collection tubes for later analysis (Figure 1A). Although FACS has been widely used for isolation of highly purified cell populations, it has been reported that FACS can also be used to sort single cells (Schulz et al., 2012). For example, BD cell sorting systems (such as the BD FACSAria III Cell Sorter) are able to isolate single cells of interest from thousands of cells in a population using up to 18 surface markers.

Since the late 1960s, remarkable advances have been made on the FACS technology including the instrumentation and the availability of a large number of highly specific antibodies. The capability of FACS technology has improved significantly from a technique limited to measuring 1–2 fluorescent species per cell to 10–15 species. The maximum number of proteins that can be simultaneously measured has progressively increased (Wu and Singh, 2012). Due to this progress, our understanding of immunology and stem cell biology has improved tremendously alongside the discovery of scores of functionally diverse cell populations (Bendall et al., 2012). It has also been reported that using the next generation cytometry, “post-fluorescence” single cell technology termed mass cytometry is theoretically capable of measuring 70–100 parameters.

Although FACS has been widely used in both basic and clinical research, there are several limiting disadvantages. First, FACS requires a huge starting number of cells (more than 10,000) in suspension. Therefore, it fails to isolate single cells from a low quantity cell population. Second, the rapid flow in the machine and non-specific fluorescent molecules can damage the viability of the sorted cells rendering the isolation a failure. Moreover, cells or cell cultures must be subjected to stimulation experiments and treated in a separate environment before FACS analysis.

### Magnetic-activated cell sorting (MACS)

Magnetic-Activated Cell Sorting (MACS) is another commonly used passive separation technique to isolate different types of cells depending on their cluster of differentiation. It has been reported that MACS is capable of isolating specific cell populations with a purity >90% purification (Miltenyi et al., 1990). MACS is based on antibodies, enzymes, lectins, or strepavidins conjugated to magnetic beads to bind specific proteins on the target cells. When a mixed population of cells is placed in an external magnetic field, the magnetic beads will activate and the labeled cells will polarize while other cells are washed out. The remaining cells can be acquired by elution after the magnetic field is turned off (Figure 1B). With this technique, the cells can be separated by charge with respect to the particular antigens. Positive separation techniques use coated magnetic beads and attract cells. The cells of interest are labeled while the unlabeled cells are discarded. In contrast, if species-specific substances are unavailable, a good choice is to use negative separation techniques which employ a cocktail of antibodies to coat untreated cells. In this case, labeled cells are discarded while unlabeled are retained (Grützkau and Radbruch, 2010).

Of the two most common affinity-based techniques for specific cell isolation, MACS technology is comparatively simple and cost-effective. However, the MACS system's obvious shortcoming lies in its initial costs in the separation magnet, and running costs including not only the price of the conjugated magnetic beads, but also replacement columns. In addition, the final purity of isolated cells in MACS devices depends on the specificity and the affinity of the antibodies used to select the target cells. It also depends on the amount of non-specific cell capture. Non-specific contamination can be from adsorption of background cells to the capturing device or their entrapment within the large excess of magnetic particles needed for labeling rare cells in large volumes. Using new materials can eliminate contamination from non-specific adsorption or entrapment of other blood cells. Another disadvantage of MACS is that it can only utilize cell surface molecules as markers for separation of live cells. Furthermore, it should be noted that MACS is far more limited than FACS because of immunomagnetic techniques that can only separate cells into positive and negative populations. High and low expression of a molecule cannot be separated while it is possible by using FACS sorting.

### Laser capture microdissection (LCM)

Laser Capture Microdissection (LCM) is an advanced technology for isolating pure cell populations or a single cell from mostly solid tissue samples on a microscope slide (Emmert-Buck et al., 1996). It can accurately and efficiently target and capture the cells of interest to fully exploit emerging molecular analytical technologies, including PCR, microarrays and proteomics (Espina et al., 2007). Today, there are two general classes of laser capture microdissection systems: infrared (IR LCM) and ultraviolet (UV LCM). The LCM system consists of an inverted microscope, a solid state near infrared laser diode, a laser control unit, a joy stick controlled microscope stage with a vacuum chuck for slide immobilization, a CCD camera, and a color monitor (Datta et al., 2015). The basic principle of LCM starts with visualizing the cells of interest through an inverted microscope, then a fixed-position, short duration and focused laser pulse is delivered to melt the thin transparent thermoplastic film on a cap above the targeted cells. The film melts and fuses with the underlying cells of choice. When the film is removed, the target cells remain bound to the film while the rest of the tissue is left behind. Finally, transfer the cells to a microcentrifuge tube containing buffer solutions required for a wide range of downstream analysis (Kummari et al., 2015; Figure 1C).

The most important advantage of LCM is its speed while maintaining precision and versatility (Fend and Raffeld, 2000). LCM provides a rapid, reliable method to procure pure populations of target cells from a wide range of cell and tissue preparations via microscopic visualization (Bonner et al., 1997). Conventional techniques for molecular analysis require dissociation of tissue. This may introduce inherent contamination problems and reduce the specificity and sensitivity to subsequent molecular analysis. On the other hand, LCM is a “no touch” technique that does not destroy adjacent tissues after initial microdissection. Morphology of both the captured cells as well as the residual tissue is well preserved and reduces the danger of tissue loss (Esposito, 2007). In addition, after removing the chosen cells, the remaining tissue on the slide is fully accessible for further capture, allowing comparative molecular analysis of adjacent cells.

The major requirement for effective LCM is correct identification of cell subpopulations or single cells in a complex tissue. Thus, the major limitation is the need to identify cells of interest through visual microscopic inspection of morphological characteristics, which in turn, requires a pathologist, cytologist, or technologist trained in cell identification (Espina et al., 2007). Another significant limitation is that the microdissected tissue section does not have a cover slip. Cover slipping would prevent physical access to the tissue surface, which is crucial to any current microdissection method. Without a cover slip, and the index matching between the mounting media and the tissue, the dry tissue section has a refractile quality, which might obscure cellular detail at high magnifications. Moreover, LCM introduces a number of technical artifacts, including slicing the cells during the preparation of tissue sections and UV damage to DNA or RNA from the laser cutting energy (Allard et al., 2004).

### Manual cell picking/micromanipulation

Manual cell picking is a simple, convenient, and efficient method for isolating single cells. Similar to LCM, manual cell picking micromanipulators also consists of an inverted microscope combined with micro-pipettes that are movable through motorized mechanical stages. Each isolated single cell can be observed and photographed under the microscope, thus enabling unbiased isolation (Figure 1D). Unlike LCM that mainly isolates single cells from sections of fixed tissue, micromanipulation plays an important role in isolating live culture cells or embryo cells.

Micromanipulation can be easily performed in an electrophysiology lab equipped with a patch clamp system. For example, after investigating neuronal function in brain slices preparations after standard whole-cell patch-clamp electrophysiological recordings, scientists would apply negative pressure through the patch pipette so that the cytosolic material containing cellular mRNA can be aspirated for further analysis (Eberwine et al., 1992; Citri et al., 2012). However, the throughput is limited and it requires highly skilled professionals to perform, it has the utility limitation when detecting complex changes.

### Microfluidics

Microfluidics is recognized as a powerful enabling technology for investigating the inherent complexity of cellular systems as it provides precise fluid control, low sample consumption, device miniaturization, low analysis cost, and easy handling of nanoliters-volumes (Whitesides, 2006; Figure 1E). Cell Sorting by a microfluidic chip can be divided into four categories: cell-affinity chromatography based microfluidic (Nagrath et al., 2007), physical characteristics of cell based microfluidic separation, immunomagnetic beads based microfluidic separation, and separation methods based on differences between dielectric properties of various cell types.

Cell-affinity chromatography based microfluidic is the most commonly used method for microfluidic chip analysis. It is based upon highly specific interactions between antigen and antibody, ligand and receptor. At the beginning of the process, the micro-channel in the chip is modified with specific antibodies capable of binding to cell surface antigen or aptamer, such as an epithelial cell adhesion molecule. Once the sample flows through the micro-channels, its cell surface antigen can bind to the specific antibodies or aptamer immobilizing the cells on the chip, while the remaining cells flow off the chip with the buffer. Finally, using a different buffer, we can elute the immobilized cells for downstream analysis. Compared to other separation methods, affinity based systems have higher specificity and sensitivity because of the recognition-binding event.

Today, microfluidics can be combined with different separation methods, such as filtration and sedimentation or affinity-based technologies like FACS and MACS. In the recent years, numerous investigations and applications in microfluidic devices have been reported, including cancer research, microbiology, single-cell analysis, stem cell research, drug discovery, and screening (Arora et al., 2010; Li et al., 2012a). Recently, microfluidic chips have been fabricated from silicon or glass, elastomer, thermosets, hydrogel, thermoplastics, and paper (Ren et al., 2013, 2014). The advantages and disadvantages of the materials used in microfluidic chips have been well-summarized previously (Ren et al., 2014). Microfluidics are used to manipulate liquids (dimensions from 1 to 1000 μm) in networks of micro-channels in a single device. At such ultralow volumes, fluids exhibit different physico-chemical properties compared to their behavior at the macro-scale (Squires and Quake, 2005). Other common fluids can be used in microfluidic devices include bacterial cell suspensions, whole blood samples, protein or antibody solutions, and various buffers.

Taking advantages of integrating cell handling and processing concurrently, microfluidic chips show potential applications in DNA sequencing (Hashimoto et al., 2007; Liu et al., 2007), protein analysis (Emrich et al., 2007), cell manipulation, and cell composition analysis (VanDijken et al., 2007; Bhagat et al., 2010). For example, Fluidigm developed a commercially available valve-based microfluidic qPCR system called the Dynamic Array™. This system advanced on providing low-volume (nanoliter) and high-throughput (thousands of PCR reactions per device) methods to the researchers and has become increasingly popular for large-scale single cell studies. Moreover, microfluidic technology has shown increasing applications in studying diversity and variations in single cell genomes, spanning from cancer biology to environmental microbiology and neurobiology. Beyond genomics applications, the scalability and small volume advantages of microfluidic methods have found applications in the measurement of intracellular and secreted proteins from single cells.

## Single cell analysis

Single cell analysis tools can be divided into three groups: genomics, transcriptomics, and proteomics. Due to next generation sequencing (NGS) technologies as well as whole genome/transcriptome amplification (WGA/WTA) approaches, a new scientific field of single cell genome studies have been established. A combination of high-throughput and multiparameter approaches is used in single cell analysis which can reflect cell to cell variability and heterogeneous differences in the individual cells. Therefore, the development of efficient single cell analysis methods requires attention. In this section, we discuss novel technologies designed for single cell analysis of genomics, transcriptomics, and proteomics (Table 2).

**Table 2:** **Techniques for single cell analyses**.

	**Methods**	**Classification**	**Throughput**	**Advantage**	**Disadvantage**	**References**
Genome	PCR^*^	LA-PCR^*^, IRS-PCR^*^, PEP-PCR^*^, DOP-PCR^*^	High	High coverage	Uneven coverage, amplification bias, allele dropout	Klein et al., 1999
	MDA^*^	None	High	Homogeneous coverage	Amplification bias, allele dropout, “chimera” structure	Spits et al., 2006
	MALBAC^*^	None	High	Homogeneous coverage	Amplification bias, allele dropout	Lu et al., 2012: Van Loo and Voet, 2014
Transcriptome	PCR-based amplification	RNA-seq, TPEA^*^, SMART^*^	High	Amplify quickly	Distort the difference	Pan, 2014
	IVT^*^	CEL-seq Quartz-seq	High	Specificity, ratio fidelity	Low efficiency	Hebenstreit, 2012; Liu et al., 2014
	Phi29 DNA polymerase	TTA^*^ PMA^*^	High	High efficient, low bias	RNA need to be selected from the gDNA	Pan et al., 2013; Liu et al., 2014
Protein	Flow cytometry	None	High	More species	Spectral overlap	Haselgrübler et al., 2014
	Microfluidic flow cytometry	None	High	Small number of cells	Dissociated cells, high skill needed	Wu and Singh, 2012
	Mass spectrometry	LDI-MS^*^, SIMS^*^ (MALDI)-MS^*^	High	Low sensitivity	No molecular labels, Femtomolar sensitivity	Haselgrübler et al., 2014; Liu et al., 2014

* PCR, Polymerase chain reaction;

* LA-PCR, linker-adapter PCR;

* IRS-PCR, Interspersed repetitive sequence PCR;

* PEP-PCR, Primer extension pre-amplification PCR;

* DOP-PCR, degenerate oligonucleotide-primed PCR;

* MDA, Multiple displacement amplification;

MALBAC, Multiple annealing and looping-based amplification cycles;

* TPEA, 3′-end amplification;

* SMART, strand-switch-mediated reverse transcription amplification;

* IVT, in vitro transcription;

TTA, Total transcript amplification;

* PMA, Phi29 mRNA amplification;

LDI-MS, Laser desorption and ionization mass spectrometry;

* SIMS, Secondary ion mass spectrometry;

* MALDI-MS, Matrix-assisted laser desorption/ionization mass spectrometry.

### Single cell genomics

Single cell genome sequencing allows us to identify chromosomal variations, such as copy number and single-nucleotide variations. It also allows us to study tumor evolution, gamete genesis, and somatic mosaicism, which is reflected in the genomic heterogeneity among a population of cells. However, in humans, it often faces the low amount of genome materials, for example, the weight of one genomic DNA is only 6 pg and each gene in the genome only has two copies in a single normal cell which is not quite enough for the current NGS use. However, amplification using traditional PCR suffers from severe biases and allelic dropout across the genome when it is applied to single cells. Therefore, a precise, unbiased amplification of the DNA is critical to single cell genome sequencing. Lots of attempts were made, mostly by modifying the traditional PCR methodology to linker-adapter PCR (LA-PCR) (Klein et al., 1999), interspersed repetitive sequence PCR (IRS-PCR), primer extension pre-amplification PCR (PEP-PCR) (Hubert et al., 1992), degenerate oligonucleotide-primed PCR (DOP-PCR) (Telenius et al., 1992), and its variant displacement DOP-PCR (D-DOP-PCR) (Langmore, 2002). For example, by using DOP-PCR, Navin and colleagues demonstrated accurate and robust determination of genome wide copy number in rearranged cancer genomes (Navin et al., 2011). This is the first report of single cell genome sequencing applied to a cancer genomic heterogeneity study. However, these methods also have some limitations in low coverage, amplification bias, and allele dropout.

The multiple displacement amplification (MDA) is the most popular method applied in genome analysis due to its high fidelity and simplicity. It can amplify DNA in a 30°C isothermal reaction with random hexamer primers and phi29 DNA polymerase. The kernel of MDA is that phi29 DNA polymerase can extend the primers with high fidelity and strong processivity, which exhibits powerful strand displacement ability during the new strand synthesis (Dean et al., 2002). The displacement process generates single stranded DNA templates, which are reprimed and extended, thereby amplifying the DNA in an isothermal reaction. Based on MDA, Xu and colleagues provided the first intratumoral genetic landscape at a single-cell level and demonstrated that clear cell renal cell carcinoma (ccRCC, the most common kidney cancer) may be more genetically complex than previously thought (Xu et al., 2012). However, MDA also suffers from strong biases and high allelic dropout rate across the genome, making the reaction vulnerable to generating “chimeras,” resulting in unwanted noise and false results.

Another new method, multiple annealing and looping-based amplification cycles (MALBAC) showed faithful copy number variation detection (Zong et al., 2012), which can amplify the genome of a single cell with high uniformity. MALBAC is based upon strand displacement pre-amplification that generates amplicons with complementary ends. Thus, the full amplicons generated in the reaction seal themselves to form loops to prevent them from being amplified again. This also ensures that each new amplicon is replicated from the original templates. Therefore, the obvious advantage of MALBAC is that it can reduce the amplification errors and biases as the starting materials of the exponential amplification are amplicon separately copied from the original template. However, it is still needed to improve the fidelity and lower the bias (Marcy et al., 2007; Wu et al., 2014).

### Single cell transcriptomics

Single cell transcriptome sequencing has recently emerged as a powerful technology for revealing differential gene expression and diverse RNA splicing patterns during early embryonic development, differentiation and reprogramming. The main application of single-cell transcriptomics is to connect a cell's genotype to phenotype. It is able to detect thousands of transcripts in various kinds of tissues and cells (Cloonan et al., 2008; Mortazavi et al., 2008). Although mRNA is not as rare as DNA in a single cell, there are still thousands of copies. This is ideal since NGS transcriptome sequencing also requires a large amount of RNA as the starting material. The mRNA from single cells needs to be reverse-transcribed to cDNA followed by cycles of PCR amplification (Sandberg, 2014). The key process in completing single cell mRNA amplification successfully is based on performing reverse transcription to double-strand DNA with high efficiency and low biases.

PCR-based amplification was first reported in single-cell transcriptome analysis of the preparation of single-cell cDNAs using cDNA microarray and RNA-seq analysis (Brady and Iscove, 1993). The disadvantage of a microarray is the low detection sensitivity that would likely miss many low-level but key transcripts. Compared to microarray analysis, RNA-seq analysis expanded the spectrum of detected genes with high accuracy and effectively increased the proportion of full-length cDNA. One advantage of PCR-based mRNA transcriptome amplification bias is that it makes the expression difference more visible between samples and any RNA starting amount can be employed. But on the other hand, it may distort the original difference when it is marginal. Several modified PCR-based methods of cDNA amplification have been developed, such as global PCR amplification (GA), 3′-end amplification (TPEA), and strand-switch-mediated reverse transcription amplification (SMART) (Pan, 2014).

*In vitro* transcription (IVT)-based amplification linear RNA amplification is the first strategy that has been used to successfully amplify RNA for molecular profiling studies, which promoted the birth of the era of single cell analysis (Liu et al., 2014). It is based on T7 RNA polymerase-mediated IVT and requires three rounds of amplification. The main advantages of the IVT strategy include its specificity, ratio fidelity, and reducing accumulation non-specific products, but has the drawback of low efficiency and a time consuming procedure.

Recently, single cell RNA amplification methods have been raised based on the Phi29 DNA polymerase (Blanco and Salas, 1984; Dean et al., 2002). This polymerase is a highly processive enzyme with strong strand displacement activity that allows for highly efficient isothermal DNA. The phi29 DNA polymerase-based transcriptome amplification method is a simple, fast and isothermal reaction (Liu et al., 2014). The primary advantage of this method is the highly efficient, low bias, and uniform nature of amplification.

Furthermore, in order to retain the spatial and temporal information of RNAs in cells, several new RNA sequencing methods have been developed, including transcriptome *in vivo* analysis (TIVA), single molecule fluorescent *in situ* hybridization (smFISH), fluorescent *in situ* RNA sequencing (FISSEQ), and so on (Lee et al., 2014; Lovatt et al., 2014). These technologies become powerful tools for unraveling longstanding biomedical questions.

### Single cell proteomics

Single cell analysis of DNA and RNA can provide qualitative information about protein expression. However, they cannot give information on protein concentration, location, post-translational modifications, or interactions with other proteins. Thus, single-cell proteomics help us obtain much more information that is crucial in cell signaling and cell to cell heterogeneity. Traditional protein analysis techniques, such as gel electrophoresis, immunoassays, chromatography, and mass spectrometry require numerous cells for analysis. Therefore, the major challenges of analyzing proteins at the single-cell level are the exceedingly small copy number of individual proteins and the lack of amplification methods. However, recent advances in multiparameter flow cytometry, microfluidics, mass spectrometry, mass cytometry, and other techniques have led to new single cell proteomics studies that could be performed with greater sensitivity and specificity.

Not only widely used in cell sorting, flow cytometry is also the most established and user-friendly method for both qualitative and quantitative multiparameter analysis of single cells. As mentioned before, by using multiparameter flow cytometry, scientists can simultaneously measure 10–15 key proteins in signaling pathways in individual cells (De Rosa et al., 2001; Perez and Nolan, 2002). In addition, in an immunological proof-of-concept study, as many as 19 separate parameters including 17 fluorescent colors and 2 physical parameters were analyzed (Perfetto et al., 2004). This strong ability has turned flow cytometry into a powerful tool to semi-quantitatively analyze pathways underlying many diseases (Irish et al., 2004; Sachs et al., 2005). The main limitation is the spectral overlap due to the broad spectral emission bands of organic fluorescent dyes. Quantum dots mitigate but do not eliminate the problem. Hence, complex correction algorithms are required for spectral deconvolution. Moreover, commercial flow cytometers use cell suspensions, which in turn allow individual interrogation of cells. The sample preparation is still done manually and therefore, requires a large numbers of cells (More than 10,000). This makes it hard to analyze small samples, such as cells recovered from a biopsy, tissue specimens or small volumes of blood.

To overcome these limitations, efforts have been made to develop microfluidic-based miniaturized flow cytometers which permit analysis of small numbers of cells (100–1000) (Lindström and Andersson-Svahn, 2010). For example, Su and colleagues developed a microscope-based label-free microfluidic cytometer. It is capable of acquiring two dimensional light scatter patterns from the smallest mature blood cells (platelets), cord blood hematopoietic stem/progenitor cells (CD34 + cells), and myeloid precursor cells (Su et al., 2011). Srivastava et al. (2009) developed an integrated microfluidic device which retro-fitted to commercial. The major advantage of this microfluidic device is its ability to perform cell culture, stimulation and sample preparation in combination with conventional fluorescence imaging and microfluidic flow cytometry to monitor immune response in macrophages. These microfluidic devices not only drastically reduced the amount of sample and reagent required, but also provided a means to perform two orthogonal modes of measurements-imaging and cytometry, in one experiment.

Mass spectrometry (MS) is the most powerful tool for protein analysis. However, MS's use for analyzing proteins in single cells is limited due to the lack of sensitivity to detect low amounts of proteins. Fractionation of the cell lysate by capillary electrophoresis (CE) prior to MS offers a good way to improve sensitivity. Recently, a format for flow cytometry has been developed that leverages the precision of mass spectrometry which is termed mass cytometry. It can uniquely enable the measurement of over 40 simultaneous cellular parameters on single cells with the throughput capacity to survey millions of cells from an individual sample (Mellors et al., 2010).

## Application of single cell analysis

The exponential growth in studies applying single cell analysis is explicitly tied to the acceptance of the technique by biologists. Single cell analysis has influenced and impacted different domains of science including cancer biology, neuroscience, and immunology and so on. It is impossible to document each of these developments. Therefore, a short overview of the fields of applications that are typically addressed by single cell analysis is presented in the research and application for cancer, brain and stem cell, etc.

### Application of single cell analysis in cancer, neuron research

Intra-tumor heterogeneity has been widely reported in numerous human cancer types. Tumors are frequently composed of individual, molecularly distinct clones that differ in their proliferation rates and metastatic potential, most critically, in their sensitivities and responses to drug treatment. Those cells that can cause distant metastases should possess unique characteristics when compared to the remaining subpopulation. Exome sequencing of single cells isolated from primary renal carcinomas showed that only 31–37% of the genetic lesions within a tumor are identical to the rest of the tumor cells (Gerlinger et al., 2012; Xu et al., 2012). Therefore, analyzing the occurrence, development and metastasis of these tumors at a single cell level provides much more detailed information on how a drug will respond to the tumor cells. It has been reported that the PIK3CA mutations were detected in primary and metastatic tumor tissues, but it is different periodically in single cells of CTCs and DTCs indicated the drug efficacy (Deng et al., 2014).

Several important types of cancer cells have been discovered, including primary tumor cells, metastatic tumor cells, cancer stem cells (CSC), circulating tumor cells (CTC), and disseminated tumor cells (DTC) (Zhang et al., 2016). CTC and DTC play a vital role in cancer dissemination, self-renewal, and distant metastases. They are being increasingly recognized for their potential utility in disease monitoring and therapeutic targeting. Many cancer patients are diagnosed with early-stage cancer with no clinical symptoms of metastasis but subsequently succumb to metastatic relapse. One important reason is that CTCs in the blood and DTCs have already reached a secondary organ but have not yet grown to become clinical metastasis. However, the CTCs are so rare among massive numbers of blood cells, as few as one cell per 10 million white blood cells and 5 billion red blood cells, that the accurate identification of CTCs turns out to be the most difficult step in the isolation process (Deng et al., 2008). In recent years, a variety of enrichment and detection techniques have been developed, making significant progress in CTC detection. For example, the CellSearch® system (Janssen Diagnostics, NJ, USA) is the first and the only technique that has been approved by the US FDA for the detection, enrichment and quantification of CTCs in peripheral whole blood samples (Riethdorf et al., 2007). This system utilizes magnets with ferrofluid nanoparticles conjugated to antibodies that target epithelial cell adhesion molecules, such as EpCAM and CD45. EpCAM is the most commonly used epithelial marker that is present on epithelial tumor cells while CD45 is an immunocyte marker that is present on many blood cells but absent in epithelial cells. Thus, the findings of EpCAM-positive and CD45-negative cells indicate the presence of CTCs. Another new immunomagnetic separation technology, called MagSweeper (Illumina), involves dipping a rotating magnetic rod with bound EpCAM antibodies in order to isolate CTCs. Then moving the magnetic rod into a new buffer to release the CTCs (Talasaz et al., 2009; Powell et al., 2012). The MagSweeper can be used reliably to extract functional human CTCs from the blood of mice inoculated with human tumor xenografts, while retaining both their tumor-initiating and metastasizing capacities (Ameri et al., 2010). This highlights the most advantageous aspect of MagSweeper is that CTCs can be completely isolated while preserving the integrity and viability of these fragile cells.

In recent years, a large number of studies have been reported using single cell analysis to analyze individual tumor cells isolated from breast cancer (Navin et al., 2011; Deng et al., 2014; Wang et al., 2014; Eirew et al., 2015), colon cancer (Zong et al., 2012; Yu et al., 2014), pancreatic adenocarcinomas (Ruiz et al., 2011), muscle-invasive bladder cancer (Li et al., 2012b), intestinal cancer (Grün et al., 2015), lung adenocarcinoma cancer (Kim et al., 2015), renal cell carcinoma (Gerlinger et al., 2012; Li et al., 2012b), and acute myeloid leukemia (Ding et al., 2012; Hughes et al., 2014; Paguirigan et al., 2015). For example, Navin and colleagues investigated copy number variation in single tumor cells using DOP WGA followed by DNA sequencing to determine cell population structure and tumor evolution patterns in a single breast tumor (Navin et al., 2011). This study provided an important breakthrough for research on tumor evolution and offered a way to assess the genetic details of tumor structure. Hou and colleagues applied MDA based single cell sequencing technology for the first time to analyze primary thrombocytosis disease (essential immature, ET) in patients at single bone marrow cell level (Hou et al., 2012). Thus, understanding tumor heterogeneity via single cell analysis is considered the biggest challenge in cancer research and if elucidated would enhance our ability to determine the best treatment options.

It is no exaggeration to say that the brain is the most complex structure in the human body. There are more than 100 billion neurons in the human brain. Each of them can make approximately 10,000 direct connections with others, totaling some 100 trillion nerve connections. This makes the brain a complicated network (Herculano-Houzel, 2009). The brain is divided into several regions. Each region consist of various morphologically and/or neurochemically distinct neurons surrounded by various types of glial cells (oligodendrocytes, microglia, and astrocytes). Additionally, distinct regions in the brain, such as areas of the cerebral cortex, hippocampus have specific functions. The cerebral cortex is responsible for many "higher-order" functions like language and information processing while the hippocampus is involved in spatial learning and memory. Increasing evidence shows that each brain region contains different types of neurons according to their location, neurotransmitter identity, connectivity, electrophysiological properties, and molecular markers. Changes of genomic content and epigenetic profiling of specific neuronal or glia subtypes are involved in the pathogenesis of neuropsychiatric diseases, such as Parkinson's and Alzheimer's diseases and autism spectrum disorders(Citri et al., 2012).

Hence there is no doubt that single cell isolation and analysis have made increasingly significant contributions to our understanding of the role that somatic genome variations play in neuronal diversity and behaviors. For example, MACS based technique has been successfully applied to isolating immature neuronal cells from a large number of embryonic zebrafish; the antibody of PSA-NCAM conjugated microbeads were used within a semi-automated dissociation process. (Welzel et al., 2015). Moreover, the MACS was also used for the isolation of embryonic spinal oligodendroglial progenitor cell populations from the rat embryonic spinal cord. By using superparamagnetic MicroBeads combined with A2B5 antibodies (a specific oligodendroglial development marker) and the Mini-MACS separator column, the oligodendroglial cells were isolated with a cell purity of 58–61% in comparison to 6–12% in an unseparated population (Cizkova et al., 2009).

Moreover, basolateral amygdala (BLA) neurons are used to activate distinct populations of the lateral central nucleus of the amygdala (CeL) neurons to either promote fear or reduce anxiety. Namburi and colleagues identified two populations of neurons in the basolateral amygdala neurons that undergo opposing synaptic changes following fear (negative emotion) or reward (positive emotion) conditioning. By using RNA-seq they identified few differentially expressed candidate genes between these two population neurons that may mediate the effects (Namburi et al., 2015). Usoskin and colleagues used comprehensive transcriptome analysis of 622 single mouse neurons from sensory system and discovered 11 fundamentally distinct types of sensory neurons. Interestingly, each neuron is associated with a different type of sensation (Usoskin et al., 2015). Even cells that appear to be morphologically similar may show marked differences in expression patterns. In neuroscience research, electrophysiological analysis combined with molecular biology within the same cell will provide convincing results for us to better understand of how changes at the molecular level are manifested in functional properties (Eberwine et al., 1992).

### Applications of single cell analysis in stem cell research

Stem cells are undifferentiated cells that are characterized as both being capable of self-renewal and having the potential to differentiate into specialized types of cells. How stem cells balance their self-renewal capacity and their ability to differentiate are central questions in stem cell research. Stem cells can be generally classified into pluripotent stem cells, which can give rise to cells of all three germ layers (the ectoderm, mesoderm, and endoderm) or tissue-specific stem cells (also referred to as somatic or adult stem cells), which play essential roles in the development of embryonic tissues and the homeostasis of adult tissues. Both of these two types of stem cells are intermingled with a variety of differentiated and intermediate cell types in the embryonic or adult tissues, forming heterogeneous populations. Therefore, isolation, analysis, and development of specific therapies that target stem cells give cancer patients hope for improvement in terms of survival and quality of life, (Li et al., 2008; Sharma et al., 2010).

Cancer stem cells (CSCs) are hypothesized to persist in tumors as a distinct population and cause relapses and metastases by forming new tumors. CSC are intrinsically more refractory to the effects of a variety of anticancer drugs possibly via enhanced drug efflux (Trumpp and Wiestler, 2008). These cells are especially resistant to therapeutic drugs. Due to the limited number of CSCs in cancer tissues, isolation and analysis CSCs are still a hard work. Single cell sequencing provides powerful tools for identifying these cells providing new insight into complex intra-tumoral heterogeneity. For example, Patel et al. (2014) used single-cell RNA sequencing to profile 672 single cells from five primary. Each tumor showed high intra-tumoral cell heterogeneity in many aspects, including copy number variations as well as cell cycle, immune response and hypoxia. By examining a set of “stemness” genes, they identified continuous, rather than discrete, stemness-related expression states among the individual cells of all five tumors, reflecting the complex stem cell states within a primary tumor. It has been suggested that CSCs are more resistant to chemo—and radiotherapy than other cells in a tumor. This could be one explanation to why most tumors relapse after therapy. Thus, understanding how cancer stem cells resist medical therapy could lead to the development of new, more efficient cancer treatments. Although the existence of these CSCs is still controversial in many cancer types, there is no doubt that CSCs have the potential to provide a foundation for new innovative treatment targeting the roots of cancer.

The neural stem cells (NSCs) in the subventricular zone (SVZ) and the subgranular zone (SGZ) of the dentate gyrus continually divide and differentiate into mature neurons and glia in the adult rodent brain (Aimone et al., 2014). Although it has been documented that endogenous NSCs can be activated to produce multiple types of progeny to contribute to brain repair after brain injury, people do not know how distinct pools of NSCs may react to brain injury and which molecules trigger injury-induced activation of NSCs. Single-cell sequencing reveals a population of dormant neural stem cells in the SVZ that become activated upon brain injury by down regulation of glycolytic metabolism and a concomitant up regulation of lineage-specific transcription factors and protein synthesis (Llorens-Bobadilla et al., 2015).

Increasing evidence shows that multiple molecularly distinct groups of stem cells that respond differently to physiological stimuli coexist in the tissues. Understanding and implementing this molecular diversity will be critical in harnessing the potential of disease treatment.

## Conclusion and outlook

The biological relevance of cell to cell variations and the high potential of single cell analysis in both basic research and clinical diagnostics have drawn the attention of the scientific community. Single cell gene expression analysis can be used for tumor cell identification; single cell DNA mutation analysis can be used for tumor cell monitoring and clinical decision making (Powell et al., 2012; Deng et al., 2014). Understanding cellular heterogeneity has been a major thrust of technological development over the past decade, resulting in an increasingly powerful suite of instrumentation, protocols, and methods for analyzing single cells at the DNA sequence, RNA expression and protein abundance levels (Kalisky et al., 2011; Wu and Singh, 2012). As remarkable examples, technical developments, and appropriate clinical solutions based on single cell analyses of CTCs and CSCs showed the promise to uncover personalized medicine to fight against cancer.

Although much progress has been made during the recent years in single cell gene analysis, live single cell isolation and molecular analyses are more favorable for global profiling of RNA expression and DNA mutation (Powell et al., 2012). We are still only beginning to face the measurement challenges of cellular heterogeneity. There is still more room for improvement in enabling new modes of analysis and improving the sensitivity, precision, speed and throughput (Lecault et al., 2012).

For single cell genomic and gene expression analyses, the greatest obstacle for direct detection of diverse genomic, transcriptomic, and epigenetic events is whether there is a sufficient amount of DNA or RNA. On the one hand, purification of high-quality nucleotides from a single sample plays a pivotal role for the following studies. A problem that is commonly faced is tube absorption which causes loss of sample materials. Low absorption material containers instead of ordinary tubes and single tube reaction analysis are recommended to reduce the loss of DNA and RNA, single cell direct PCR/RT-PCR without nucleotide isolation are also often used. Another problem is the low replication efficiency of secondary structure DNA sequences. Methods for current single cell sequencing still have relatively high technical noise. It is acceptable when studying highly expressed genes, but the biological variations of genes that are expressed at low levels may be masked. Thus, the efficiency of reverse transcription and PCR amplification should be urgently improved. On the other hand, this problem could be overcome by the third-generation sequencing platforms, which are based on sequencing single molecules and real-time signal monitoring (Schadt et al., 2010; Liu et al., 2012). Within third-generation sequencing technology, no amplification is required and it also overcomes the issue of PCR amplification bias. However, the detection sensitivity, accuracy of sequencing reads, sample handling, recovery, and sequence assembly still need to be further improved.

Protein analysis is far more challenging than nucleic acid analysis. Undoubtedly, the complexity of the proteome, lack of amplification methods and highly specific high-affinity probes make protein analysis technically demanding. Because the cell contents are highly diluted after lysis, high affinity probes (not only monoclonal antibodies), and highly sensitive detection methods are needed to detect low abundance proteins and post-translational modifications.

To summarize, single cell analysis now stands poised to illuminate this new layer of biological complexity under normal development and disease conditions. Considering the rapid progress in either the development of single cell isolation or analysis technology, many of the problems mentioned above will be solved in the near future. Nevertheless, further developments and interdisciplinary co-operative work between technologists, scientists, and clinicians will be necessary. In the distant future, we expect that the single cell techniques will become a powerful tool to unravel longstanding questions in both biological research and clinical diagnostics.

## Author contributions

GD and HX conceived the structure of the manuscript; PH and WZ wrote the manuscript; GD and HX read, edited, and approved the manuscript; Mr. Brian Deng (Stanford University) helped the discussion and correction of English writing.

### Conflict of interest statement

The authors declare that the research was conducted in the absence of any commercial or financial relationships that could be construed as a potential conflict of interest. The reviewer ASB and handling Editor declared their shared affiliation, and the handling Editor states that the process nevertheless met the standards of a fair and objective review.
